# An improved method for isolation of RNA from bone

**DOI:** 10.1186/1472-6750-12-5

**Published:** 2012-01-19

**Authors:** Lauren E Carter, Gail Kilroy, Jeffrey M Gimble, Z Elizabeth Floyd

**Affiliations:** 1 Ubiquitin Biology Laboratory, Pennington Biomedical Research Center, 6400 Perkins Road, Baton Rouge, LA 70808 USA; 2 Stem Cell Biology Laboratory, Pennington Biomedical Research Center, 6400 Perkins Road, Baton Rouge, LA 70808 USA

## Abstract

**Background:**

Bone physiology is increasingly appreciated as an important contributor to metabolic disorders such as type 2 diabetes. However, progress in understanding the role of bone in determining metabolic health is hampered by the well-described difficulty of obtaining high quality RNA from bone for gene expression analysis using the currently available approaches.

**Results:**

We developed a simple approach to isolate bone RNA that combines pulverizing the bone and the phenol-guanidinium based RNA extraction in a single step while maintaining near-freezing temperatures. This single step method increases the yield of high quality RNA by eight-fold, with RNA integrity numbers ranging from 6.7 to 9.2.

**Conclusions:**

Our streamlined approach substantially increases the yield of high-quality RNA from bone tissue while facilitating safe and efficient processing of multiple samples using readily available platforms. The RNA obtained from this method is suitable for use in gene expression analysis in real-time quantitative PCR, microarray, and next generation sequencing applications.

## Background

Obtaining intact, high quality RNA is an essential step in analyzing gene expression. This step is particularly challenging in bone, which contains low numbers of cells embedded within a highly mineralized tissue. As the endocrine functions of bone [1] and the relationship between bone and adipose physiology [2] becomes increasingly apparent, the need to isolate high quality RNA for gene expression analysis in bone using the current genome-wide sequencing technologies will gain more importance.

**Figure 1:**
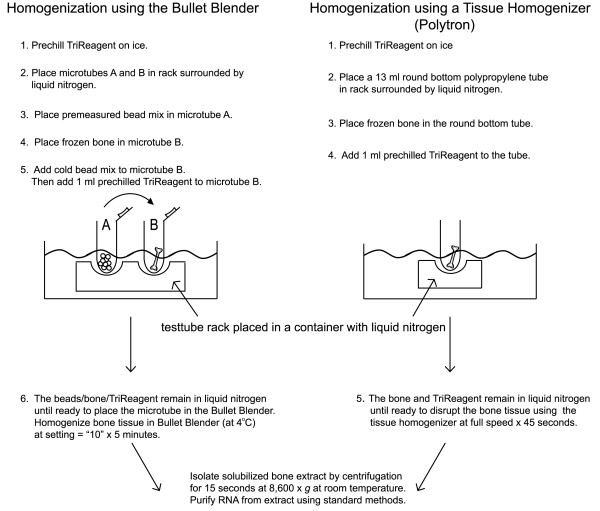
**Overview of the One-Step Method for Isolation of RNA from Bone**.

Current methods for isolating RNA from bone use multiple steps in which the frozen bone is wrapped in foil, refrozen in liquid nitrogen and ground into a powder using a hammer [3] or ground using a mortar and pestle containing liquid nitrogen[4-6]. The powdered bone is then transferred to a second container for extraction of the RNA using a phenol-guanidinium-based reagent. While these approaches support extraction of RNA from bone, the multiple steps introduce the possibility of sample loss and the potential for cross-contamination. In addition, this approach does not lend itself to efficiently processing multiple samples. Herein, we report a one-step method for extracting RNA from bone (Figure 1) that consistently yields high-quality RNA suitable for gene expression analysis using the currently available technologies and is readily adaptable to various platforms.

## Results

### Extracting RNA from bone in a single step

All animal studies using C57BL/6J male mice were performed with approval from the Pennington Biomedical Research Center Institutional Care and Use Committee using mice purchased from Jackson Laboratory (Bar Harbor, ME). Femur bones were harvested from five month old male mice that were fed a defined low fat (D12450B, Research Diets, Inc. New Brunswick, NJ) or high fat diet (D12451, Research Diets, Inc) beginning at four weeks of age. Any attached tissue was quickly removed from the bone using a scalpel before the bone was snap frozen in liquid nitrogen. The bone was stored at -80°C for up to two months before isolating the RNA. For RNA isolation, each bone sample was transferred from -80°C storage to liquid nitrogen until it was divided into two equal portions. To divide the bone, the femurs were cut using diagonal pliers (6 inch/solid joint, TopMost) that are available at hardware stores. One half of the bone was added to a microtube (1.5 ml Eppendorf Safe-Lock) that was prechilled by placing the microtube in a rack surrounded by liquid nitrogen. The remaining half of the bone was immediately returned to the liquid nitrogen and then stored at -80°C.

To facilitate processing multiple samples, we used the Bullet Blender (Next Advance) centrifuge technology that homogenizes tissue using bead disruption of the tissue. A four hour incubation of previously isolated liver RNA with RNase-free beads or untreated beads (Next Advance) demonstrated that RNA is not degraded when in contact with the untreated beads (Figure 1A). We then attempted to isolate RNA from bone using the Bullet Blender, which was kept at 4°C in a cold room.

**Figure 2:**
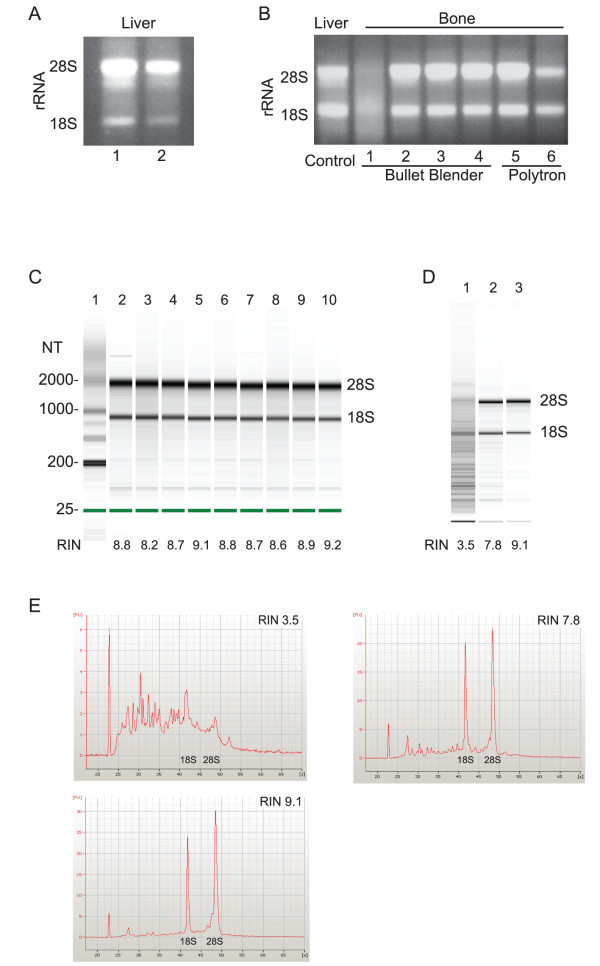
**Extracting High Quality RNA from Bone in a Single Step**. (A) Isolated liver RNA was incubated with RNase free beads (lane 1) or untreated beads (lane 2) provided by the manufacturer (Next Advance) for four hours before analyzing the 18S and 28S rRNA bands using agarose gel chromatography. Subsequent experiments with the Bullet Blender were carried out using untreated beads. (B) Bone RNA was homogenized in near freezing conditions using the Bullet Blender centrifuge (lanes 2-4) or a Polytron (lanes 5-6). The results are compared to bone RNA isolated using standard homogenization conditions (lane 1) and intact liver RNA that was previously isolated (control). (C) The RNA Integrity Number (RIN) for RNA homogenized in near freezing conditions was determined using the Agilent RNA 6000 Nano LabChip Kit and the Agilent 2100 Bioanalyzer. RNA with a RIN = 7 or greater is suitable for microarray analysis of gene expression. (D) The maximum RIN for the two-step approach (lane 2) is compared to the maximum RIN obtained in the one-step approach (lane 3) and each RIN is compared to the RIN of the degraded RNA sample (lane 1) shown in the agarose gel in lane 1, Figure 2B. (E) The electropherograms associated with the samples shown in (D).

The bone was added to a prechilled microtube containing the beads recommended by the manufacturer for RNA isolation (~50 μl stainless steel blend, 6 × 3.2 mm stainless steel, and 1 × 4.2 mm stainless steel) followed by the addition of 1 ml TriReagent (Molecular Research Center). The bone was homogenized in the Bullet Blender centrifuge for five minutes at a speed setting of "ten". The solubilized bone extract was separated from the beads and pulverized bone material by centrifugation at 8,600 × *g *for fifteen seconds at room temperature. Following centrifugation, the RNA was isolated from the resulting extract using an RNeasy Mini Kit (Qiagen) at room temperature. Using this approach, the RNA was consistently degraded (Figure 2B, **lane 1**). Next, we altered our method to maintain the bone at a near freezing temperature during the entire homogenization procedure (Figure 1). The beads were added to the microtube, which was placed on ice to prechill the microtube and beads. The TriReagent was also prechilled on ice. The bottom of the microtube containing the beads was dipped into liquid nitrogen to freeze the beads before pouring the cold bead mix into the microtube containing the frozen bone. One ml of the prechilled TriReagent was added to the microtube containing the bone and beads. Each microtube containing the beads, bone, and TriReagent was kept in the rack surrounded by liquid nitrogen while the remaining samples were prepared. Each tube was allowed to warm slightly with handling before placing the tube into the Bullet Blender to avoid fracturing the microtube during homogenization of the bone. As before, the bone was homogenized in the Bullet Blender centrifuge (kept in a cold room at 4°C) for five minutes at a speed setting of "ten" followed by isolation of the solubilized bone extract by centrifugation for fifteen seconds at 8,600 × *g *at room temperature. Using this approach, none of the isolated RNA was degraded. Representative samples are shown in Figure 2B, lanes 2-4.

To determine if our approach is applicable to other methods of homogenizing the bone tissue, we repeated the procedure using a Polytron (Kinematic PT 3100, 7 mm tip) to homogenize the tissue (Figure 2B, **lane 5, 6**). The reagents and bone were handled as described for the Bullet Blender with the exception that beads are not required when using the Polytron. Homogenization in the Polytron was carried out for 45 seconds at full speed in 13 ml round bottom polypropylene tubes (Sarstedt), which were prechilled in a rack surrounded by liquid nitrogen (Figure 1).

We compared this approach to currently used methods that employ separate steps to pulverize the bone and extract the RNA [3-6]. While the one-step approach has the advantage of maintaining the bone and reagents at near-freezing conditions during the homogenization, the currently used methods can be carried out using a simple mortar and pestle rather than the specialized equipment required for the one-step approach. To compare the quality of RNA obtained with the two approaches, we repeated the RNA isolation using a multi-step method that pulverizes the bone independently of the phenol-guanidinium based RNA extraction. We placed the frozen bone in prechilled sterilized foil followed by refreezing the bone and foil briefly in liquid nitrogen. The foil-wrapped bone was placed in a mortar that was prechilled in liquid nitrogen and the bone was pulverized using a pestle until the bone was powdered [3]. The powdered bone tissue was transferred to a microtube for addition of the TriReagent (1 ml) and extraction and isolation of the RNA. The RNA extract was separated from the powdered bone material by centrifugation at 8,600 × *g *for fifteen seconds at room temperature followed by isolation of the RNA using an RNeasy Mini Kit (Qiagen) at room temperature.

### RNA Quality and Yield

RNA yield was determined by the absorbance at 260 nm (Table 1) and using the Agilent RNA 6000 Nano LabChip Kit and the Agilent 2100 Bioanalyzer (Agilent Technologies). The one-step approach yielded eight-fold higher amounts of RNA with less variation in the yield when compared to the two-step approach (Table 1). In addition to assaying the RNA for intact 28S and 18S rRNA (Figure 2A, B) using agarose gel electrophoresis, we determined the quality of the isolated RNA using the Agilent 2100 Bioanalyzer. Quantification of the RNA quality is reported in an RNA Integrity Number (RIN) that takes into account the entire electrophoretic RNA trace produced in the analysis [7]. A RIN of greater than or equal to seven indicates the RNA is suitable for high stringency applications [7]. As shown in Figure 2C, the representative samples have a RIN greater than 8.0 and the average RIN for thirty-two samples analyzed was 8.28 (Table 1). Although the RIN for RNA isolated using the two-step approach was as high as 7.8 (Figure 2D), the average was 5.65 (Table 1), indicating variability in the quality of RNA obtained. However, recent data indicates that RNA degradation does not interfere with gene expression analysis when the RIN is greater than 5 [8]. The electropherograms in Figure 2E further illustrate the relationship between the RIN and the extent of RNA degradation.

**Table 1:** RNA Yield and Quality Using a One-Step Approach to Extract RNA from Bone

	Yield (μg)	A260/280	A260/230	RIN
one-step	26.0 -/+ 7.76	2.09 -/+ 0.015	2.17 -/+ 0.163	8.28 -/+ 0.87
two-step	3.17 -/+ 3.04	1.91 -/+ 0.155	1.09 -/+0.760	5.65 -/+ 2.15

RNA yield is reported based on the absorbance at 260 nm using a Nanodrop spectrophotometer and confirmed using the Agilent RNA 6000 Nano LabChip Kit and the Agilent 2100 Bioanalyzer. RNA quality is reported as the ratio of the absorbance at 260 nm and 280 nm along with the ratio of the absorbance at 260 nm and 230 nm as well as the RNA Integrity Number based on the entire electrophoretic RNA sample trace produced using the Agilent 2100 Bioanalyzer. Each value is reported at the mean -/+ standard deviation.

**Figure 3:**
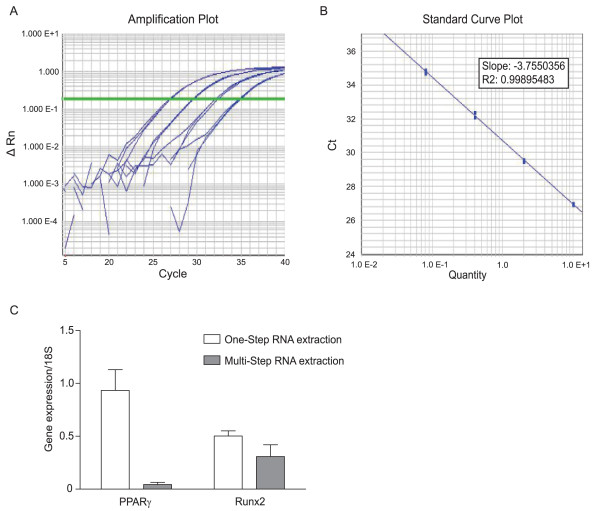
**The Isolated Bone RNA Supports Gene Expression Analysis using Real-time qRT-PCR**. One μg of the isolated RNA was reverse transcribed using Multiscribe Reverse Transcriptase (Applied Biosystems) with random primers at 37°C for 2 hours. Gene expression of the bone specific transcription factor Runt-related transcription factor 2 (Cbfa1/Runx2) and the adipocyte-specific transcription factor Peroxisome Proliferator-Activated Receptor Gamma (PPARγ) was analyzed via real-time PCR performed with TaqMan chemistry using the 7900 Real-time PCR system and universal cycling conditions. The Runx2 and PPARγ TaqMan primer-probe pairs were obtained from Applied Biosystems. (A) The amplification plot and (B) standard curve generated for Runx2. (C) Comparison of the gene expression of PPARγ and Runx2 from bone RNA isolated from the one-step approach versus multi-step methods. The bone was obtained from an animal fed a high fat diet for 4 months.

To demonstrate the suitability of the bone RNA isolated via a one-step approach for quantitative real-time RT-PCR, 1 microgram of RNA was reversed transcribed using Multiscribe Reverse Transcriptase (Applied Biosystems) with random primers at 37°C for 2 hour. Real-time PCR was performed with TaqMan chemistry (Applied Biosystems) using the 7900 Real-Time PCR system (Applied Biosystems) and universal cycling conditions (50°C for 2 minutes; 95°C for 10 minutes; 40 cycles of 95°C for 15 seconds and 60°C for 1 minute; followed by 95°C for 15 seconds, 60°C for 15 seconds and 95°C for 15 seconds). All results were normalized to a 18S rRNA expression. The gene expression of the bone specific transcription factor Runt-related transcription factor 2 (Cbfa1/Runx2; ABI Mm00501580_m1, amplicon length 129 bases) [9] and the adipocyte-specific transcription factor Peroxisome Proliferator-Activated Receptor Gamma (PPARγ; Mm01184323_m1, amplicon length 101 bases) was analyzed (Figure 3). The sample was obtained from an animal fed a high fat diet for 4 months. Bone and fat cells share a common origin and activation of PPARγ with diet-induced obesity is associated with decreased bone density, increased expression of PPARγ in bone, increased bone marrow adipogenesis and decreased expression of Runx2 [10]. The results in Figure 3C indicate the one-step method of isolating RNA from bone yields significantly higher levels of PPARγ in bone when compared to the currently used approach. The increased level of PPARγ expression in the bone measured after the one-step RNA extraction is consistent with the effects of a high fat diet on adipogenesis in bone.

## Discussion

In this study, we present a simple method for isolating bone RNA that relies on homogenizing the bone tissue using reagents and materials that are maintained at temperatures near freezing during a single step that combines fragmentation of the bone with extraction of the RNA. This practical and low cost method is readily adaptable to any homogenization equipment that is compatible with cooling the reagents and materials to near freezing. The results demonstrate that a single step approach consistently gives higher yields of intact RNA with RNA Integrity Numbers necessary to support demanding gene expression applications when compared to current methods.

## Conclusions

This approach consolidates extraction of RNA from bone into a single step that allows routine isolation of high quality bone RNA from multiple samples without sample loss or possible cross contamination. This approach will facilitate studies designed to investigate changes in bone gene expression by ensuring a high yield of RNA suitable for demanding gene expression applications.

## Authors' contributions

LEC participated in discussions to develop the method, isolated the RNA, carried out the agarose gel chromatography and real-time RT-PCR assays and participated in drafting the manuscript. LEC and ZEF constructed the figures for the manuscript. GK participated in discussions to develop the method, assisted LEC in the real-time RT-PCR, carried out the analysis of RNA quality using the Agilent Bioanalyzer and edited the manuscript. JMG participated in discussions regarding the method development and edited the manuscript. ZEF participated in discussions regarding the method and wrote the manuscript. All authors read and approved the final manuscript.
